# Particulate matter exposure in construction sites is associated with health effects in workers

**DOI:** 10.3389/fpubh.2023.1130620

**Published:** 2023-03-07

**Authors:** Eghbal Sekhavati, Reza Jalilzadeh Yengejeh

**Affiliations:** Department of Environmental Engineering, Ahvaz Branch, Islamic Azad University, Ahvaz, Iran

**Keywords:** respiratory exposure, suspended particulate matters, cancer risk, non-cancer risk, construction industry

## Abstract

**Background:**

Exposure to suspended particulate matters (PMs) at high concentrations, mainly observed in the construction workplace, is found to be a risk factor for major health outcomes. The present study was conducted to investigate the degree of exposure to suspended PMs in different stages of construction of the buildings and the health risk associated with the exposure in Lar, Fars, Iran.

**Methods:**

In this cross-sectional study, two construction sites were selected in Lar. Cancer and non-cancer health risks of exposure to PM_2.5_ and PM_10_ were assessed using the US Environmental Protection Agency method in three-dimensions: inhalation, digestion, and dermal absorption. The hazard quotient (HQ) and total cancer risk (TCR) were considered as parameters for risk analysis.

**Results:**

The highest level of non-cancer risk for workers in the concentrations of PM_2.5_ and PM_10_ particles in the drilling process were determined to be 2.97 × 10^−1^ and 8.52 × 10^−2^, respectively. In the cancer risk analysis, PM_10_ concentrations were estimated to be at the highest level (1.7 × 10^−7^) in the drilling process and the lowest level (4.29 × 10^−8^) in the facilities process. For suspended PM_2.5_, it was an unacceptable risk level in all processes, except for the implementation of facilities.

**Conclusion:**

These results show that the construction industry, especially in developing countries such as Iran, needs better management to maintain the health of construction workers.

## Introduction

Nowadays, activities at construction sites are considered a global health concern. In a work environment, employees can face numerous health risks, including Biological Hazards (1, 2) Chemical Hazards (3–7), Physical Hazards (8–10), and ergonomic hazards (11, 12) in these sites that could be harmful for the health of workers; these factors have made construction activities among the riskiest industries in the world (13, 14). Besides, these activities are also increasing which makes the health threat even more (15); however, due to difficulty for assessment, dangers, and uncleanness, the construction industry has not been under evaluation sufficiently and there are still issues related to health and safety (16).

Suspended particulate matters (PM) including cement, dust, gypsum, etc. in the air among these construction site related factors that workers are dealing with every day of their work (17, 18). Exposure to these suspended PM at high concentrations, mainly observed in the construction workplace, is found to be a risk factor for major health outcomes (19, 20). Epidemiological studies have shown that this exposure could be associated with several acute and chronic respiratory system sequels and cardiovascular diseases (21–23). In 2010, the International Agency for Research on Cancer classified suspended PMs as group 1 carcinogens, i.e., proven to be carcinogenic to humans (24). Furthermore, there is a higher risk of mortality among those who are frequently exposed to PMs (25–27).

It has been showed that the potential health effects attributed to exposure to PMs are highly dependent on the nature matters suspended in the environment and their size (28, 29). Besides, the hazardous dose of exposure to PMs differs based on these two parameters, the matter type and its size (16). There is a large population of workers in the construction industry in Iran. These populations is confronted with a high rate of burden (30). However, there is a limited body of evidence regarding the extent of this health concern and the status of PMs these workers are exposed to in less developed regions of Iran such as Lar, a city in southern Iran, with a high load of construction activities. Health assessment of these effects is significant in implementing strategies to improve the health of workers working in this industry. Therefore, the present study was conducted to investigate the degree of exposure to suspended PM in different stages of construction of the buildings for construction workers and the health risk associated with the exposure in Lar.

## Materials and methods

This cross-sectional study was conducted on two main construction sites in Lar city in southern Iran with a population of about 221 thousand people over 2020. The statistical population in this research included 374 workers of these two construction sites which determined based on Cochran formula. The Sampling error percentage was determined based on the following formula introduced by García-Closas et al. (31):


$$F_{1}.Nsample=\;\frac{\left(ta^{2}.CV2\right)}{d^{2}}$$

Where t_a_ is equal to 1.96 at a significance level of 5%. Also, considering the infinite degree of freedom, CV will be the percentage of the coefficient of variation (the ratio of standard deviation to the average) of the mass tram of suspended dust particles in each of the construction sites. Also d is the ratio of the allowed or required error (10%).

The type of activity in the studied construction workshops is the same. The activities of demolishing, Excavation, concreting, and moving construction materials are among the factors affecting the emission of dust. The two studied workshops are active for the construction of 300 residential units. The major tasks of the workers in construction sites are marking, carrying construction materials, excavation, concreting, brick masonry, roof laying, flooring and finishing.

After estimating the adequacy of the sample (89 construction worker), a sample of air in construction sites was gathered in a regular grid at intervals of 3 × 3 m after calibration of the sampling device. Sampling of dust particles was done at every point, from a height of 180 cm above the ground. The TES 5200 Particle Mass Counter direct reading particle measuring device was used to evaluate the number density in both cumulative and class or differential modes 2 for particles with aerodynamic diameters of 0.5, 0.7, 1, 2.5, 4, 5, 7, and 10 micrometers per particle per liter. Particle mass density with aerodynamic diameters of 1, 2.5, 4.5, 7, 10, and 10–100 micrometers in micrograms per cubic meter was read using this instrument.

In terms of particle size, PM10, PM7, PM4, PM2.5, and PM1 had the highest density in terms of μg/m^3^, respectively In order to determine the mass density of the total suspended particles, all these parameters were measured. PM_x_ is defined as particles with an aerodynamic diameter less than x micrometers. Total suspended particulate (TSP) refers to the totality of small solid matter released.

The basis of the device's performance is measuring the angular dispersion or dispersion of light waves caused by a laser diode due to the passage of suspended particles of different dimensions. Calibration was done in the device, using a standard zero filter. In this case, the plastic interface contains a standard filter which placed in the area of the air inlet of the sampling device so the numbers of mass density and particles on the display screen changes to zero. By sampling a construction site, the sampler was recalibrated to Sampling from another site will reduce the measurement error. All samplings were done in such a way as to represent the same environmental conditions, mass density and number of particles during the work shift. The mass density of the total particles compared to the Threshold Limit Values (TLV).

### Health risk evaluation process

We used the US Environmental Protection Agency (EPA) method for assessing of non-cancer and cancer health risk of exposure to PM_2.5_ and PM^10^ in the construction sites (32). For health risk analysis, in the first step, we exposed the workers to the particles in three-dimensions: inhalation through the nose, digestion through the mouth, and skin absorption by the particles adhering to the skin. In the second step, the daily exposure dose (D) was calculated separately for each of the exposure methods according to Formulas (2)–(4) and their sum according to Formula (5) (33).


$$\begin{array}{l} F_{2}.\text{Ding = }(\text{C}\times {\text{R}}_{\text{ing}}\times \text{EF}\times \text{ED}\times \text{CF})\text{/}(\text{BW}\times \text{AT}).\\ F_{3}.\text{Dinh = }(\text{C}\times {\text{R}}_{\text{inh}}\times \text{EF}\times \text{ED})\text{/}(\text{BW}\times \text{AT}\times \text{PEF}).\\ F_{4}.\text{Ddermal = }(\text{C}\times \text{SA}\times \text{SL}\times \text{ABS}\times \text{EF}\times \text{ED}\times \text{CF})\text{/}(\text{BW}\times \text{AT}).\\ F_{5}.{\text{ADD = D}}_{\text{ing}}{\text{+D}}_{\text{inh}}{\text{+D}}_{\text{dermal}.} \end{array}$$

Where, the parameter C represents the mean mass density of PM_2.5_ and PM^10^ (in mg/m^3^), D_ing_ is daily exposure dose by ingestion (mg/kg/day), D_inh_ is daily exposure dose by inhalation (mg/kg/day), D_dermal_ daily exposure dose through dermal absorption (mg/kg/day), ADD is the average daily dose (mg/kg/day), BW is the average body weight considered to be 70 kg, R_ing_ is the swallowing rate, which is considered 100 mg/day for workers, R_inh_ is the inhalation rate for workers which is considered to be 20 m^3^/day, PEF is Particle Emission Factor, which is 1.36 × 109 m3/kg, SA is the area of skin in contact with airborne particles. The value of this parameter is 5,700 cm^2^, SL is a skin adhesion factor equal to 0.07 mg/m^3^, EF is the frequency of exposure and its amount is equal to 180 days per year, ED is the workers' exposure time, which equals to 30 years, AT is the mean exposure for cancer risks with is equal to 70 × 180 days and for non-cancer risks ED × EF days, ABS is the skin absorption factor which is considered 0.001 (without units), CF is the conversion factor, the amount of which is equal to 1 × 10^−6^ mg/kg.

Finally, the hazard quotient (HQ) was calculated as a parameter for the non-cancer effects of PM_10_ and PM_2.5_. The HQ parameter represents the ratio of the concentration of pollutants in the environment to its reference dose (RfD), which is calculated according to Formula (6):


$$F_{6}.\text{HQ = }{\text{D}}_{\text{ing}}{\text{+D}}_{\text{inh}}{\text{+D}}_{\text{dermal}}\text{/R}fD.$$

In this formula, component RfD is the reference exposure dose. The RfD value for PM_10_ is 1.1 × 2^−10^ mg/kg/day, and that for PM_2.5_ is 8.5 × 10^−4^. If the values obtained from the calculation of the HQ parameter are <1, there is no significant risk of creating non-cancer risks. If the values of this index are >1, non-cancer risks are possible.

To calculate the cancer risks of exposure to PM_10_, we used Formula (7):


$$F_{7}.\text{R = ADD}\times \text{SF}.$$

Where, the SF parameter represents the cancer slope factor. The above formula is simplified in the form of Formula (8):


$$F_{8}.\text{TotalCancerRisk}(\text{TCR}){\text{=Risk}}_{\text{ing}}{\text{+Risk}}_{\text{inh}}\text{+}\;{\text{Risk}}_{\text{dermal}}$$

The cancer slope factor for PM_10_ is equal to 2×10^−6^ mg/kg/day. Cancer slope factor values for PM_2.5_ are not provided; therefore, we could not calculate cancer risk.

TCR between 10^−6^ (one in 1,000,000) and 10^−4^ (one in 10,000) is acceptable, while risks higher than 10^−4^ are unacceptable.

Statistical Package for Social Sciences (SPSS) version 19 (CA, The United States of America) was used in this study for data cleaning and statistical analyses. Continued variables were presented using mean and standard deviation (SD). For categorical variables, frequency and relative frequency were calculated. Chi-square test and one-way analysis of variance (ANOVA) were applied for analyses. Pearson correlation coefficient was used to determine whether there was a correlation between exposures and health risks.

## Results

Based on the size of the study sites, 92 points were identified for data gathering. Examination of mass and number of particles showed that the average total particle density was 5,545.53 μg/m^3^. The highest average suspended PMs were found in the excavation process, 2,170.43 μg/m^3^, and the lowest was in the facilities process, 565.88 μg/m^3^. It should be noted that in the construction process, the values of suspended PM concentration are variable, and these values are related to the time-space average of each point at the time of measurement. The average mass concentration of suspended PMs in different stages of building construction in terms of μg/m^3^ is presented in Table 1.

**Table 1:** Average mass concentration of suspended particulate matters in different stages of construction in terms of micrograms per cubic meter.

**Suspended particulate matters**	**Roof implementation**		**Concreting**		**Excavation and foundation**		**Facilities**	
	**Mean (**μ**g/m**^3^**)**	**SD**	**Mean (**μ**g/m**^3^**)**	**SD**	**Mean (**μ**g/m**^3^**)**	**SD**	**Mean (**μ**g/m**^3^**)**	**SD**
PM_1_	35.76	6.09	32.28	5.16	45.54	4.89	17.19	4.19
PM_2.5_	142.69	23.11	124.76	9.28	176.13	15.27	39.48	6.54
PM_4_	156.74	26.74	137.9	16.33	215.98	14.73	41.76	9.63
PM_7_	451.08	117.08	303.45	47.98	552.75	88.31	106.92	16.87
PM_10_	437.25	65.37	325.07	89.91	653.3	103.21	165.47	36.38
TSP	375.66	44.15	286.58	56.19	526.73	54.55	195.06	35.52
Total	1,599.18	282.54	1,210.04	224.85	2,170.43	280.96	565.88	109.13

PMs, particulate matters; SD, standard deviation; TSP, total suspended particulate.

**Figure 1:**
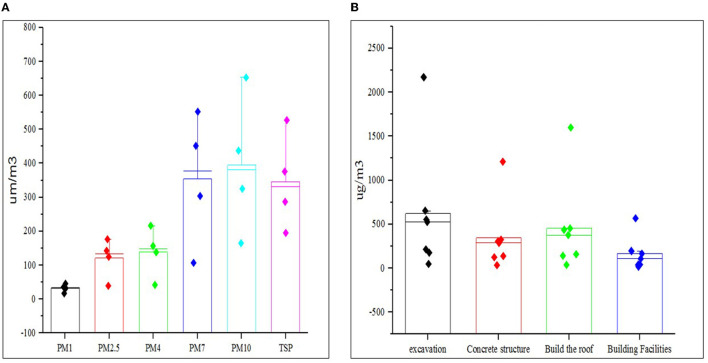
**(A, B)** Comparison of average total concentration of suspended particulate matters in construction processes at the sampling site and by particle dimensions.

A comparison of the average total concentration of suspended PMs in construction processes at the sampling site and based on particle dimensions are presented in Figures 1A, B, respectively. The maximum number of particles in the drilling process was 3,236,567 particles per liter in the dissociated state and 2,744,312 particles per liter in the cumulative state. In total, 39.1% of the total particles were in the drilling process, 21.8% in the concrete structure process, 28.8% in the roof construction and 10.2% in the facilities process.

The results of ANOVA showed that there was a significant difference between the amounts of suspended PMs such as PM_1_ (*p* = 0.011), PM_2.5_ (*p* = 0.025), PM_4_ (*p* = 0.032), PM_7_ (*p* = 0.035), PM_10_ (*p* = 0.031), and total suspended PM (*p* = 0.016) in the air in the four building processes. These showed that the larger the dimensions of suspended PM measured in the air, the higher the number of particles.

After measuring the concentration of suspended particulate matter in various building construction processes, cancer and non-cancer risk of exposure for construction workers using the EPA method was assessed. As shown in Table 2, the amount of suspended PM measured in the excavation and drilling process was higher than the amount in other processes. This was 9.37 × 10^−4^ mg/kg/day for PM_10_ and 1.79 × 10^−4^ mg/kg/day for PM_2.5_. Also, the daily exposure dose values for cancer risks, the level of PM10 intake in the excavation process was estimated to be 4.02 × 10^−4^ mg/kg/day.

**Table 2:** Daily exposure dose values for non-cancer and cancer PM_2.5_ and PM_10_ risks.

**Particles**	**Process**	**D_ingest_**	**D_inhalation_**	**D_dermal_**	**ADD (mg/kg.day)**
**Daily dose for non-cancer PM**_2.5_ **and PM**_10_ **risks**					
PM_2.5_	Excavation and foundation	2.51 × 10^−4^	1.85 × 10^−7^	2.35 × 10^−5^	2.52 × 10^−4^
	Concreting	1.78 × 10^−4^	1.31 × 10^−7^	1.06 × 10^−5^	1.79 × 10^−4^
	Roof implementation	2.03 × 10^−4^	1.49 × 10^−7^	0.89 × 10^−5^	2.04 × 10^−4^
	Facilities	0.56 × 10^−4^	4.14 × 10^−8^	4.79 × 10^−6^	5.66 × 10^−5^
PM_10_	Excavation and foundation	9.33 × 10^−4^	6.86 × 10^−7^	1.13 × 10^−4^	9.37 × 10^−4^
	Concreting	4.64 × 10^−4^	3.41 × 10^−7^	5.52 × 10^−5^	4.66 × 10^−4^
	Roof implementation	6.24 × 10^−4^	4.59 × 10^−7^	6.5 × 10^−4^	6.27 × 10^−4^
	Facilities	2.36 × 10^−4^	1.73 × 10^−7^	1.28 × 10^−5^	2.37 × 10^−4^
**Daily dose for cancer PM**_10_ **risks**					
PM_10_	Excavation and foundation	4 × 10^−4^	2.94 × 10^−7^	1.6 × 10^−6^	4.02 × 10^−4^
	Concreting	1.99 × 10^−4^	1.46 × 10^−7^	7.94 × 10^−7^	2 × 10^−4^
	Roof implementation	2.68 × 10^−4^	1.97 × 10^−7^	1.06 × 10^−6^	2.69 × 10^−4^
	Facilities	1.01 × 10^−4^	7.45 × 10^−8^	4.04 × 10^−7^	1.02 × 10^−4^

D, dose; D_ing_, daily exposure dose by ingestion; D_inh_, daily exposure dose by inhalation; D_dermal_, daily exposure dose through dermal absorption; ADD, average daily dose.

The results of the non-cancer risk analysis showed that the highest level of risk for PM_2.5_ suspended particles in the drilling process was 2.97 × 10^−1^, and the lowest level was 6.66 × 10^−2^ in the facilities process. Also, in assessing the level of non-cancer suspended particulate matter PM_10_, it was found that the highest level of non-cancer risk in the drilling process was 8.52 × 10^−2^ and the lowest level of risk in the facilities process was 2.15 × 10^−2^. It should be noted that, in general, due to the nature of suspended particulate matters < 2.5 μm, their level of non-cancer health risk was higher than PM_10_. The calculated risk levels were significantly different between PM_10_ and PM_2.5_ (*p* < 0.05) (Table 3).

**Table 3:** Risk potential values and risk index for non-cancer risks of PM_2.5_ and PM_10_.

**Particles**	**Process**	**HQ_ingest_**	**HQ_dermal_**	**HQ_inhalation_**	**HI**
PM_2.5_	Excavation and foundation	2.96 × 10^−1^	1.24 × 10^−3^	2.97 × 10^−1^	2.52 × 10^−4^
	Concreting	2.1 × 10^−1^	8.14 × 10^−4^	2.21 × 10^−1^	1.79 × 10^−4^
	Roof implementation	2.4 × 10^−1^	1.2 × 10^−3^	2.4 × 10^−1^	2.04 × 10^−4^
	Facilities	6.6 × 10^−2^	3.06 × 10^−4^	6.66 × 10^−2^	5.66 × 10^−5^
PM_10_	Excavation and foundation	8.5 × 10^−2^	3.12 × 10^−4^	8.52 × 10^−2^	9.37 × 10^−4^
	Concreting	4.2 × 10^−2^	2.07 × 10^−4^	4.24 × 10^−2^	4.66 × 10^−4^
	Roof implementation	5.7 × 10^−2^	2.15 × 10^−4^	5.7 × 10^−2^	6.27 × 10^−4^
	Facilities	2.1 × 10^−2^	9 × 10^−5^	2.15 × 10^−2^	2.37 × 10^−4^

HQ, hazard quotient.

In the cancer risk analysis for PM_10_, it was found that the highest level of risk in the drilling process was 1.7 × 10^−7^, and the lowest level of risk in the facilities process was 4.29 × 10^−8^. Based on the fact that in the non-cancer risk index the reference for PM_2.5_ was 1 × 10^−1^ and it was 1 × 10^−2^ for PM_10_, it can be concluded that the level of suspended particulate matter PM_2.5_ in drilling processes, metal frames and implementation of the roof was unacceptable, and in the implementation of the facility, it was at an acceptable level. Also, the level of risk of exposure to suspended particulate matter PM_10_ was unacceptable for construction workers in all processes. Moreover, the exposure level for the suspended particulate matter PM_10_ was 1 × 10^−6^. Hence, PM_10_ cancer risk was acceptable for construction workers in all processes (Table 4).

**Table 4:** Risk potential values and risk index for PM_10_ cancer risks.

**Particles**	**Process**	**R_ingest_**	**R_dermal_**	**R_inhalation_**	**TCR**
PM_10_	Excavation and foundation	1.69 × 10^−7^	6.67 × 10^−10^	1.24 × 10^−10^	1.7 × 10^−7^
	Concreting	8.44 × 10^−8^	3.36 × 10^−10^	6.2 × 10^−11^	8.48 × 10^−8^
	Roof implementation	1.13 × 10^−7^	4.53 × 10^−10^	8.3 × 10^−11^	1.14 × 10^−7^
	Facilities	4.29 × 10^−8^	1.71 × 10^−10^	3.16 × 10^−11^	5.66 × 10^−5^

R_ingest_, risk through ingestion; R_dermal_, risk though dermal absorption; R_inhalation_, risk though inhalation; TCR, total cancer risk.

## Discussion

This study was conducted to investigate the degree of exposure to suspended PM, PM_2.5_, and PM_10_ for construction workers on each level of the building, separately. In developing countries, such as Iran, where the rate of construction activities is high and the workers' health is of lower importance due to economic reasons, performing research activities to determine the exposure and risk of cancer is of crucial importance. Overall, our results showed that there were significant levels of health effects, especially non-cancer risk among construction workers. Cheriyan and Choi, a study in line with our result showed construction sites are one of the primary and simple sources of suspended PM pollution (34) and there should be routine monitoring and effective measures to decrease the health effect attributed to them.

This study showed that the drilling process could result in high exposure of construction workers to PMs. Besides, we found that even routine activities of the worker while drilling makes them exposed to the high concentrations of suspended PMs which could be associated with serious outcomes. Previous studies suggest that the exposure could be reduced regarding both the amount of PMs and the time of exposure, if efficient methods with proper equipment are used by the workers (35). Therefore, to reduce the health effects of PMs Exposure, the worker should be instructed and better machines should be prepared for them. It should also be important to pay attention to the presence of PM as PM_10_ in these places in drilling workers. One of the issues that needed to be considered in future research is the type of suspended PMs based on their origin.

We also found that the level of risk of suspended PM_2.5_ except for facilities was unacceptable in building processes. The exposure level of suspended PM_10_ is unacceptable for construction workers in all processes. Consistent with our study, in a similar study conducted by Tavakole et al., cancer risk associated with exposure to silicate PMs in a construction site was assessed and they reported that the risk was unacceptable. In a cross-sectional study by Lumens and Spee, it was also shown that the level of health risk of exposure to quartz particles in a building site was unacceptable (36). According the results of Cherian and Choi, the concentration of PM_10_ and PM_2.5_ remain 30 and 8 fold higher than their respective 24-h exposure standards (37). In the study of Luo et al. it was determined that Wearing protective masks and spraying systems could effectively reduce health risks by 67.54% (38).

The presence of an unacceptable level of risk for cancer- related health issues highlights the immediate need for improvement in the health management of construction workers in this city. This study had one important limitation. There was no control group for workers and exposure to PM in this study. One of the most advantages of the present study using a mass density index to demonstrate the exposure to suspended PMs, given that the recommended limits for suspended particulate matter are per unit mass (such as milligrams per cubic meter). The variety of activities and the exposure time of workers to PMs were among other limitations that may affect the accuracy of some results. Conducting clinical studies on workers who are exposed to suspended particles can complement the present research.

## Conclusion

This study showed that workers' exposure to airborne particles at construction sites is an important health risk. Construction workers are faced with various stages of building construction, the most important process being the excavation and excavation stages. These results show that the construction industry, especially in developing countries such as Iran, needs better management to maintain the health of construction workers.

## Data availability statement

The original contributions presented in the study are included in the article/supplementary material, further inquiries can be directed to the corresponding author.

## Author contributions

ES and RY make substantial contributions to conception, design, acquisition of data, analysis and interpretation of data, drafting of this study, and the guarantors of this work. ES participate in drafting the article or revising it. RY gives final approval of the version to be submitted and any revised version. Both authors have read and approved the manuscript.
